# Parallelised online biomass monitoring in shake flasks enables efficient strain and carbon source dependent growth characterisation of *Saccharomycescerevisiae*

**DOI:** 10.1186/s12934-016-0526-3

**Published:** 2016-07-25

**Authors:** Stefan Bruder, Mara Reifenrath, Thomas Thomik, Eckhard Boles, Konrad Herzog

**Affiliations:** 1 Institute of Molecular Biosciences, Goethe University Frankfurt, 60438 Frankfurt am Main, Germany; 2 aquila biolabs GmbH, 52499 Baesweiler, Germany

**Keywords:** Shake flask, Biomass monitoring, Online monitoring, Backscattering, *S. cerevisiae*, Parallelisation, Bioprocess automation, Metabolic shift, Diauxic effects

## Abstract

**Background:**

Baker’s yeast, *Saccharomyces cerevisiae*, as one of the most often used workhorses in biotechnology has been developed into a huge family of application optimised strains in the last decades. Increasing numbers of strains render their characterisation highly challenging, even with the simple methods of growth-based analytics. Here we present a new sensor system for the automated, non-invasive and parallelisable monitoring of biomass in continuously shaken shake flask cultures, called CGQ (“cell growth quantifier”). The CGQ implements a dynamic approach of backscattered light measurement, allowing for efficient and accurate growth-based strain characterisation, as exemplarily demonstrated for the four most commonly used laboratory and industrial yeast strains, BY4741, W303-1A, CEN.PK2-1C and Ethanol Red.

**Results:**

Growth experiments revealed distinct carbon source utilisation differences between the investigated *S. cerevisiae* strains. Phenomena such as diauxic shifts, morphological changes and oxygen limitations were clearly observable in the growth curves. A strictly monotonic non-linear correlation of OD_600_ and the CGQ’s backscattered light intensities was found, with strain-to-strain as well as growth-phase related differences. The CGQ measurements showed high resolution, sensitivity and smoothness even below an OD_600_ of 0.2 and were furthermore characterised by low background noise and signal drift in combination with high reproducibility.

**Conclusions:**

With the CGQ, shake flask fermentations can be automatically monitored regarding biomass and growth rates with high resolution and parallelisation. This makes the CGQ a valuable tool for growth-based strain characterisation and development. The exceptionally high resolution allows for the identification of distinct metabolic differences and shifts as well as for morphologic changes. Applications that will benefit from that kind of automatized biomass monitoring include, amongst many others, the characterization of deregulated native or integrated heterologous pathways, the fast detection of co-fermentation as well as the realisation of rational and growth-data driven evolutionary engineering approaches.

## Background

Baker’s yeast, *Saccharomyces cerevisiae*, is one of the most often used workhorses not only in basic research but also for biotechnological and industrial applications. During decades of research and development, a tremendous number of individual strains have been isolated, selected and constructed. Moreover, due to the recent advances in strain construction (reviewed in [1, 2]), the number of new, distinct strains will increase even faster. Therefore, it is becoming more and more challenging to characterize the huge number of strains in an exact and timely manner. Besides specific assays, growth-based analytics remain as one of the simplest methods.

Shake flasks are the most popular bioreactor system for small scale fermentations [3] due to their ease of use, their cost efficiency and their capability to be utilised in parallelised screening experiments [4]. Monitoring the growth of shake flask cultures has been carried out traditionally by manual sampling and offline biomass analysis. However, this process has become insufficient for modern data driven bioprocess monitoring purposes due to low data density, the requirement for invasive sampling and the lack of automatization and parallelisation. Over the last decade, online monitoring techniques for shake flask fermentations were developed, which are based on the measurement of oxygen and pH [5–7]. While these two parameters can offer valuable information within the bioprocess development and optimization workflow, they are not capable of completely replacing the traditional biomass based monitoring. In recent years, some non-invasive biomass sensors for shake flasks and shaken microtiter plates have been introduced [8–10]. The common basis of these sensors is the optical measurement of fermentation broth turbidity, which is then correlated to offline biomass values, such as OD_600_ or cell dry weight (CDW). Continuously shaken bioreactors are inherently characterised by a rapidly rotating, dynamic liquid distribution. All of the aforementioned sensor systems seek to eliminate this major challenge of robust and reproducible biomass estimation in shaken systems by various means (e.g. acceleration coupling, flash lamp synchronisation, stopped shaking movement), thus trying to turn the dynamic nature of shaken bioreactors into a static measurement setup. While simplifying the measurement task itself, this approach comes at the cost of measurement resolution, sensitivity and robustness as well as at the cost of a reduced liquid-dynamical application range regarding filling level, shaking speed, shaking diameter and baffling. Especially fermentations with low filling volumes and high shaking speed cannot be biomass-monitored sufficiently using the “static measurement”-approach.

Here we present a new sensor system for the automated, non-invasive and parallelisable estimation of biomass in continuously shaken shake flask cultures, called CGQ (“cell growth quantifier”). In contrast to the aforementioned devices, the CGQ implements a dynamic approach of backscattered light measurement. Based on high-speed data acquisition, a series of scattering intensity points is created that reflects the dynamic fluid distribution and allows for highly accurate measurements under any shaking condition. The advantages of this new dynamic biomass estimation approach are presented here exemplarily for the individual characterization of growth parameters of the four most commonly used laboratory and industrial yeast strains, namely BY4741, W303-1A, CEN.PK2-1C and Ethanol Red.

BY4741 is one of the parental strains of the international systematic *S. cerevisiae* gene disruption project [11]. A collection of more than 6000 strains each deleted in a single open reading frame (ORF) within the BY background allows fast characterization for various purposes [12]. The strain is a direct descendant of the original S288c isolate [11, 13]. This non-natural derived strain, generated from various crosses among three baking and three natural strains from rotting fruit, was the first eukaryotic organism whose genome has been sequenced and is still used as reference genome [14, 15]. It was selected for minimal nutritional requirements, a non-flocculent phenotype and should be formerly used for isolation of biochemical mutants [16]. Furthermore it does not show invasive or pseudohyphal growth [17]. A major drawback of S288c and its derivatives are a high petite frequency due to mitochondrial genome instability [18].

Proteomic studies and microarray analysis indicated that W303 derivatives maintain high similarity to S288c. In fact, this strain is the main but not exclusive ancestor [19, 20]. Recently, the genome of W303-K6001, a direct descendant of W303-1A was sequenced and barely differs (8133 positions) from S288c [15]. Differences between BY4741 and W303-1A are especially evident in physiological parameters such as cell size and volume, in their relative plasma-membrane potential as well as in their tolerance to alkali-metal cations [21]. Furthermore, it is proposed that senescent W303 cells develop larger cell volumes due to larger vacuoles [22]. W303-1A is widely used in physiology research [23], for instance as model organism for regulation of oxidative stress response [24], in ageing research [15] or in cell separation because of its (haploid) bipolar bud site selection [25]. Popularity is also owed to the adenine auxotrophy, which probably leads to glutathione-mediated accumulation of red pigments in the vacuole under adenine-limiting conditions [26] and is used in white red mutant screening [27, 28]. The assumption, that adenine auxotrophy provides a neutral background for physiology studies were recently controversially discussed since a direct impact of adenine depletion on cell size, trehalose content and subsequent stress tolerance could be shown [23].

The CEN.PK strains were developed for studying metabolic fluxes and functional gene analysis [29]. The descendant of CEN.PK2-1C was created by mating a slightly flocculent strain, which showed fast growth during chemostat cultivations with another non-flocculent laboratory strain. The resulting derivatives were screened for fast growth rates in complete and defined media under aerobic and anaerobic conditions, high mating, sporulation and transformation efficiency and no more flocculation [30]. Despite hypersensitivity to sodium and, in particular lithium ions [31], as well as deviations in cAMP signalling [32, 33], strains from the CEN.PK family are widely used in fundamental and applied yeast research. They have been thoroughly compared with other laboratory strains [34, 35]. The genome of CEN.PK113-7D was recently sequenced [36, 37].

The diploid industrial strain Ethanol Red is widely used in industry for first-generation bioethanol production due to its good performance in fed-batch fermentations on molasses and tolerance to high ethanol yields. Recently, it has been also established for laboratory studies [38]. Due to its high tolerance against inhibitory compounds in lignocellulosic hydrolysates, a crucial trait for second-generation bioethanol production [39], it is now even used in metabolic and evolutionary engineering approaches for pentose fermentations [38, 40]. Ethanol Red was chosen for our study as a representative diploid strain. In opposite to haploid strains, size and volume of diploid cells are clearly enhanced [41] and might therefore influence growth measurements as determined by optical densities of the newly developed CGQ system.

To demonstrate the characteristics and benefits of the new CGQ system as compared to other methods, the various strains were inoculated in shake flask cultures with media with different kind and concentrations of carbon sources, and growth was monitored by using the CGQ system. While yeast cells ferment glucose nearly completely to ethanol, galactose is partially consumed by respiration. The disaccharide maltose is taken up by a proton symport mechanism and hydrolysed intracellularly into glucose (reviewed by [42]). Furthermore, ethanol and glycerol were tested as purely respiratory carbon sources. The CGQ system proved to be superior in terms of resolution, growth detection limits, reproducibility, signal stability and smoothness as well as in terms of usability, parallelisability and handling.

## Results and discussion

### The cell growth quantifier (CGQ)

The CGQ has been developed to allow accurate and parallelisable monitoring of biomass in shake flasks in a fully automated and non-invasive manner. Special focus during the development was put on the measurement range with the goal of building a system able to monitor the complete bioprocess, from inoculation (OD_600_ < 0.5) to harvesting (OD_600_ > 50). Currently there are three other devices available, which can serve as a technical benchmark for the CGQ system, namely the SFR Vario (Presens GmbH, Germany), the OD-Monitor (TAITEC, Japan) and the OD-Scanner (BugLab LLC, USA).

**Fig. 1:**
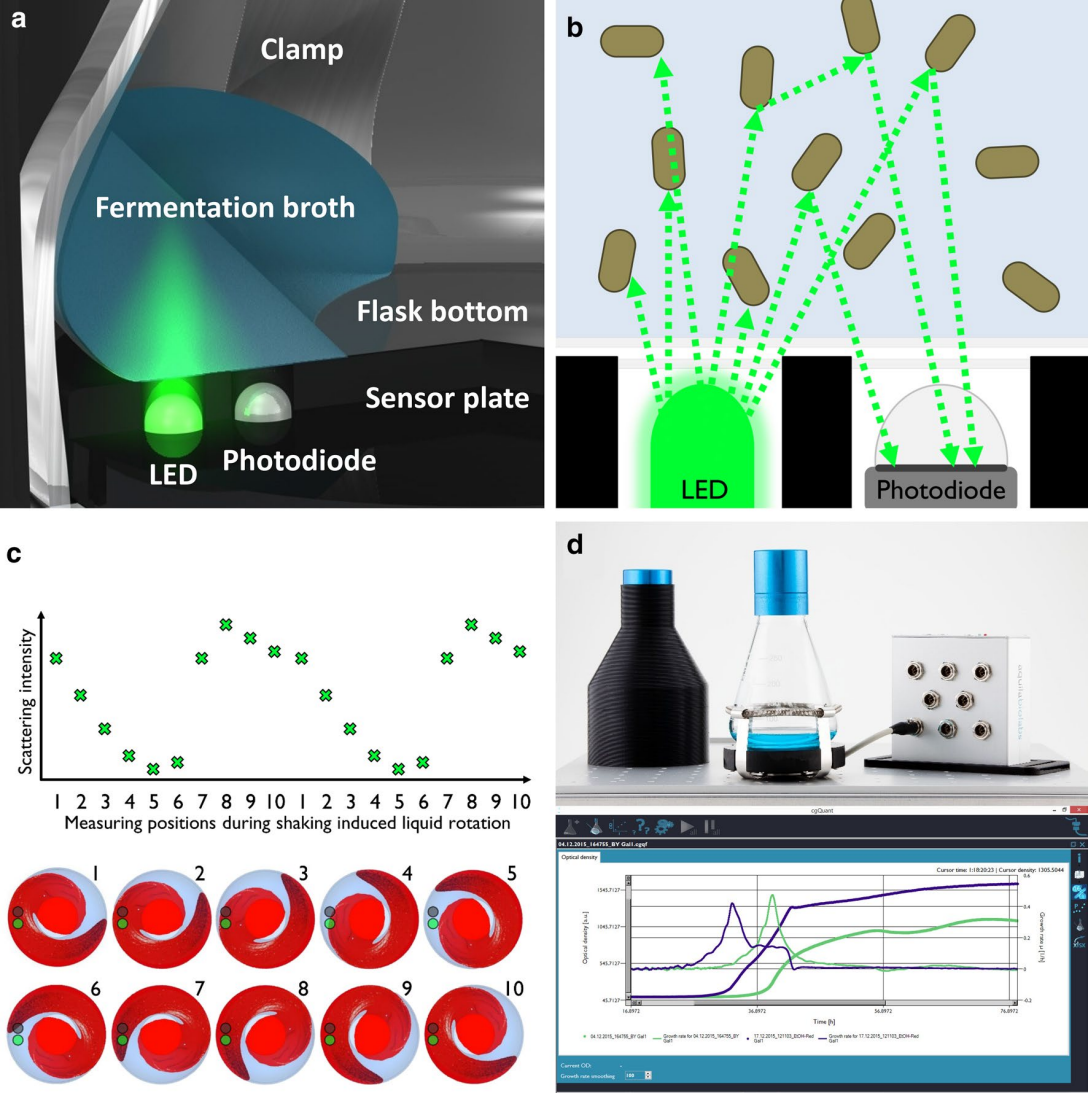
The CGQ (cell growth quantifier) as an automated system for highly parallelisable and non-invasive real-time monitoring of biomass concentration in shake flasks. **a** CGQ sensing architecture with a sensor plate measuring through the shake flask bottom. **b** Biomass monitoring by backscattered light detection. **c** Simplified scheme of continuous high-speed scattering measurements of the CGQ. The liquid movement above light source and sensor results in a periodic raw signal as the basis for robust biomass monitoring. **d** The CGQ, modularly built up of the sensor plates under each flask, the black light shielding cover, the base station and the CGQuant software

The CGQ implements the well-known technique of light scattering with light source and sensor mounted directly into the spring clamp under the shake flask (Fig. 1a). Depending on the current biomass concentration inside the flask, different amounts of light are scattered towards the sensor (Fig. 1b). Backscattered light measurements are known to exhibit a wide dynamic range and are also the basis for SFR Vario and OD-Scanner. In contrast, the OD-Monitor performs transmission measurements, which strongly reduces the upper detection limit to OD_600_ < 10 and which requires relatively high and thus in case of oxygen limited processes unfavourable filling volumes of about 20 % [43].

In addition to the dynamic range requirements of typical bioprocesses, fully automated measurements under continuous shaking are favourable to avoid effects like sedimentation, poor aeration and mixing, anaerobic metabolic stress and to reduce the scientist’s manual workload. Similar to the CGQ, the SFR Vario and the OD-Monitor allow for such measurements, while the OD-Scanner requires the flask to be taken off the shaker and to be hold still for the measurement period by the operator.

Automated biomass monitoring in shake flasks requires some kind of handling the highly dynamic liquid distribution in a continuously shaken environment. Current approaches turn the dynamic nature of shaken bioreactors into a static measurement setup. The simplest method is to stop the shaking movement as required for the OD-Scanner operation. Other methods include accelerometric synchronisation in combination with a median filter (SFR Vario) [8] and a light path choice for minimal fluid fluctuations in combination with averaging (OD-Monitor) [44].

In contrast to the aforementioned static measurement methods, the CGQ for the first time implements a dynamic approach of biomass monitoring in a continuously shaken system. For this purpose it collects millions of single backscatter readings at a dynamic measurement frequency >500 kHz, which together form periodic raw signal series (simplified shown in Fig. 1c). As the actual biomass concentration can be assumed to be constant during the few seconds of raw data acquisition, the periodic raw data series are essentially a function of the liquid height above the sensor. The CGQ collects a complete “image” of the dynamic liquid distribution over several subsequent shaking movement periods instead of simplifying the fluid dynamics to one single static measurement. From the resulting huge amount of data at a macroscopic fermentation time point, robust backscattering readings are then calculated for almost each kind of liquid distribution observable in typical shake flask experiments, even at highest shaking speeds and extremely low filling levels (i.e. 350 rpm at <5 % filling volume for optimal oxygen transfer rates). Furthermore, the data-driven CGQ technology provides real-time biomass data at high resolution and smoothness, without the need for smoothing and filtering (as described in [8]), where subsequent measurement delays of 5–10 min are introduced, which can cause significant systematic biomass underestimation errors.

In order to avoid the interference of external light sources with the highly sensitive CGQ measurements and to allow for accurate biomass monitoring at typical low inoculation cell densities, each monitored flask is darkened with a light shielding cover as shown in Fig. 1d.

One of the most favourable shake flask properties is their parallelisability. The currently available devices do not support such experimental setups and can be operated in parallel with only four flasks (SFR Vario, OD-Monitor) or even only one flasks (OD-Scanner) at the same time. The CGQ allows for fully parallelised biomass measurements in up to 8 or 16 flasks, all connected to one base station (see Fig. 1d), so that each flask on a typical shaker tray is monitored. Additionally the CGQ requires not much more space on the tray than the standard spring clamp, which furthermore contributes to its applicability in highly parallelised shake flask experiments.

Out of all currently existing solutions for non-invasive biomass monitoring in shake flasks, the CGQ system was identified as the optimal choice for an efficient triplicate based parallel evaluation of four different *S. cerevisiae* strains grown on various carbon sources due to its comparably small size, high parallelisability and dynamic range, real-time data availability and smoothness and its applicability under almost any cultivation condition. With the bundled CGQuant software (see screenshot in Fig. 1d), all data analysis tasks, including the calibration, the comparison, annotation and documentation of data sets and the calculation of growth rates (see “Methods” section) could be accomplished intuitively and satisfactorily.

### Evaluation of online biomass data in comparison to offline data for CEN.PK2-1C on SCD medium

**Fig. 2:**
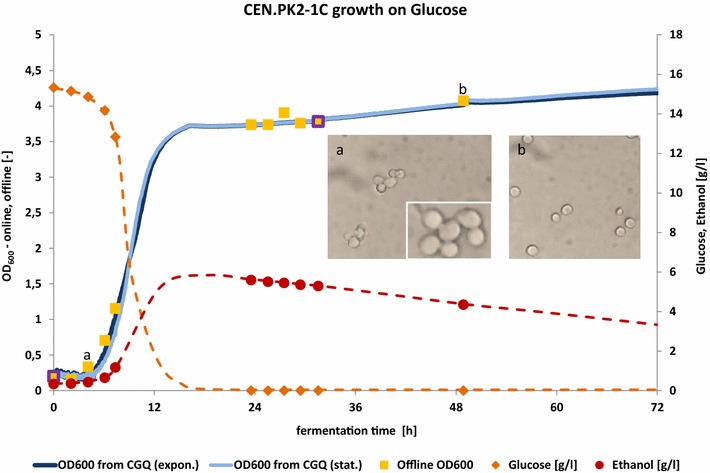
CGQ growth curves in the context of typical offline data for CEN.PK2-1C grown on glucose as carbon source. Comparison of CGQ growth measurements and offline OD_600_, HPLC data of glucose and ethanol as well as microscopic images for *S. cerevisiae* CEN.PK2-1C on SC media with 1.5 % glucose. CGQ-backscatter readings were converted into OD_600_ using calibration files either for stationary cells or for exponentially growing cells. *Yellow squares* with *purple edges* indicate offline OD_600_ values being taken by the CGQuant software for correlation data refinement. Microscopic images depict representative snapshots of the current cell morphologies and sizes at the respective sampling time (*a* and* b*, as marked in the chart) and were all collected under the same magnification. The *inset* in microscopic image* a* depicts additionally 1.5-fold magnified cells from the same droplet during excessive budding

In order to evaluate the general validity of CGQ-derived online biomass data in the context of typically collected offline parameters, CEN.PK2-1C was grown on synthetic complete medium with d-glucose and samples were taken at various time points throughout the fermentation process. CGQ backscattered light intensities were converted into OD_600_ values by CGQuant using two different calibration files for CEN.PK2-1C as depicted in Fig. 5b. The samples were analysed by offline OD_600_ measurements, HPLC and microscopy. All these results are depicted in Fig. 2. Offline and online biomass data are in good agreement, thus demonstrating the general applicability of backscattered light measurements as an alternative for offline biomass determination. The well-known Crabtree-effect of *S. cerevisiae* being grown on glucose as carbon source can be clearly identified solely on the basis of the online biomass data. Here the initial rapid growth phase on glucose accompanied by ethanol formation took about 16 h and was followed by the typical metabolic shift to a much slower growth on the basis of respiratory ethanol metabolization. These observations are strongly supported by the HPLC-derived ethanol and glucose concentrations and are in good agreement with the data published by Anderlei et al. [45] for a comparable experimental setup.

### Growth measurements of various yeast strains on different carbon sources

**Fig. 3:**
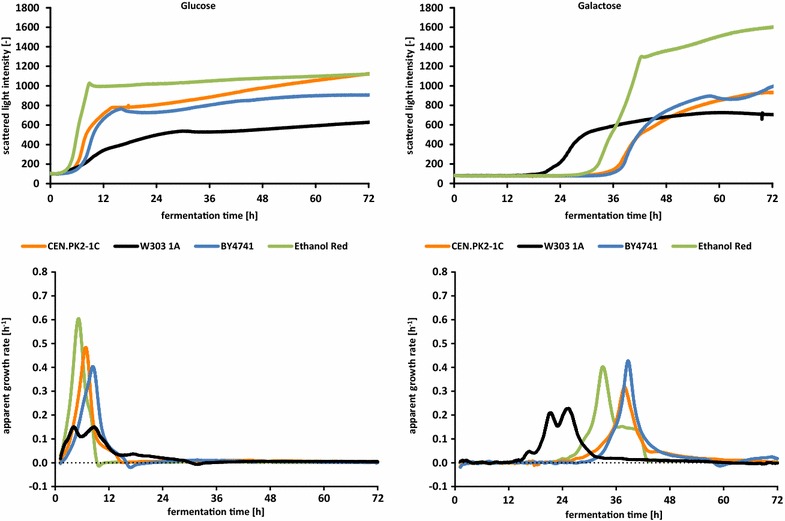
CGQ growth curves and apparent growth rates on d-glucose and d-galactose media. Comparison of CGQ growth measurements for *S. cerevisiae* strains CEN.PK2-1C, W303-1A, BY4741 and Ethanol Red on SC media with 2 % d-glucose (*left*) and 2 % d-galactose (*right*). *Top* measurements of scattered light intensities. *Bottom* calculation of apparent growth rates

**Fig. 4:**
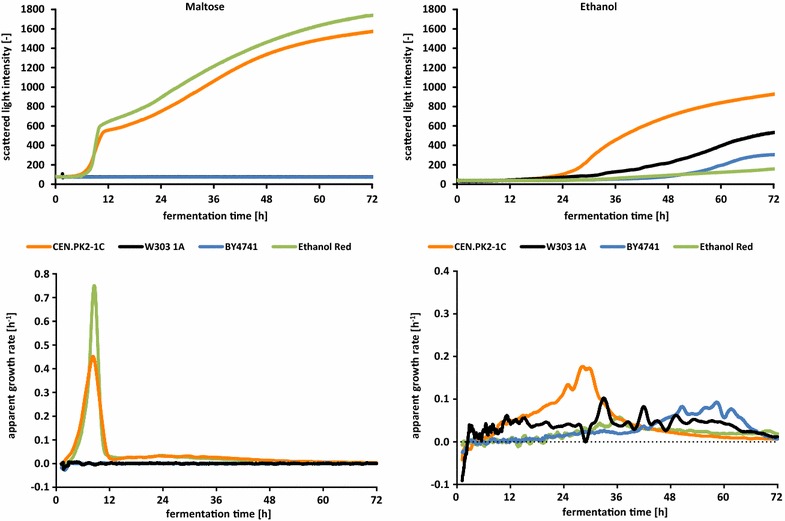
CGQ growth curves and apparent growth rates on maltose and ethanol media. Comparison of CGQ growth measurements for *S. cerevisiae* strains CEN.PK2-1C, W303-1A, BY4741 and Ethanol Red on SC media with 1 % maltose (*left*) and 2 % ethanol (*right*). *Top* measurements of scattered light intensities. *Bottom* calculation of apparent growth rates

To demonstrate the properties of the CGQ technology, four different yeast strains, BY4741, W303, CEN.PK2-1C and Ethanol Red, were grown in synthetic complete (SC) medium with different carbon sources: d-glucose, d-galactose, maltose or ethanol. Therefore, pre-cultures were inoculated in 500 mL flasks, containing YEPD2% medium, until reaching OD_600_ of 1–2. Subsequently, cultures were washed and inoculated with a starting OD_600_ of 0.2 in 300 mL flasks, containing SC medium and the particular carbon source (“Cultivation conditions” section). Growth and apparent growth rate were followed by using the CGQ (Figs. 3, 4).

On glucose medium the fastest increase of scattered light intensity, the highest intensities as well as the highest growth rate could be observed for Ethanol Red (Fig. 3). Metabolization of galactose provided the highest growth rate for strain BY4741, while the highest intensities were reached by Ethanol Red. Due to the cultivation of the pre-cultures on glucose-containing medium, long lag-phases could be shown for all strains on galactose. Interestingly, W303-1A revealed the shortest lag-phase. On maltose medium (Fig. 4), Ethanol Red reached the highest intensities and growth rate, CEN.PK2-1C was slightly slower, but no growth could be observed for BY4741 as well as W303-1A. Whether the non-existing growth of both derivatives of S288C on maltose could be due to the progenitor’s inability for maltose utilization [16, 46] or due to other reasons cannot finally be concluded by these exemplarily intended experiments.

Another interesting observation which could be nicely demonstrated with the CGQ technology was the formation of double peaks within the apparent growth rates, as shown here for the growth rates of W303-1A on glucose and galactose (Fig. 3). Whether the monitored observations are primary derived from physiological (for instance alterations in glucose repression) or morphological alterations could be a subject of further studies.

On ethanol, only a very slow growth can be seen for Ethanol Red which probably is due to its selection for high ethanol production rates and titers [38] (Fig. 4). The highest intensities and growth rate were reached by CEN.PK2-1C. No growth could be detected for all four strains on synthetic glycerol medium (data not shown). Inconsistencies with the work of Swinnen et al. [47], where prototrophic versions of the laboratory strains CEN.PK, W303, and S288c exhibited growth in a different synthetic glycerol medium (according to [48]) after supplementation of nucleobases and amino acids, could be due to the different handling of pre-cultures, different pH-values, different composition of both media or different genotypic characters.

### Additional observations

Ethanol or also small parts of fermentative carbon sources are metabolized by *S. cerevisiae* using respiratory pathways. Under the chosen shaking and aeration conditions, most aerobic cultivations can be expected to become oxygen limited as soon as a certain biomass concentration is reached. This has been shown within a comparable experimental setup [45] for *S. cerevisiae DSM70449* on YEPD2% by online monitoring of oxygen transfer rates in conjunction with offline glucose and ethanol concentrations. Indeed, those oxygen limitations are observable in real-time biomass data (as shown in [49]). With growth rates being limited by the maximal oxygen transfer rate, the biomass concentration becomes a linear function of the fermentation time. As clearly depicted in Figs. 2 and 3 for growth on glucose as primary carbon source, a strictly linear biomass increase can be observed for the respiratory ethanol metabolization phase after the diauxic shift that follows the consumption of glucose.

**Fig. 5:**
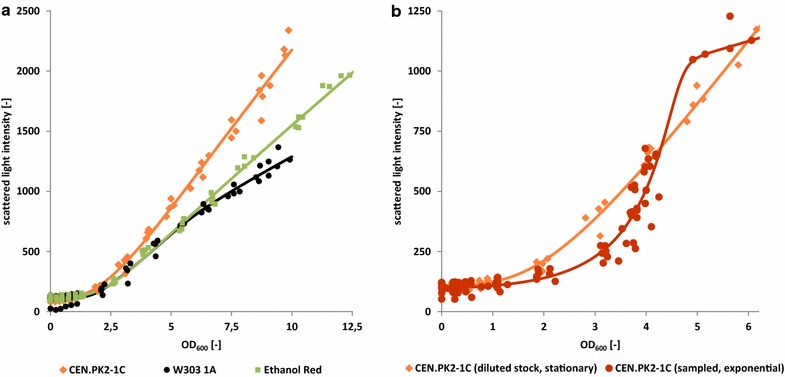
Correlation of scattered light measurements and OD_600_. **a** Comparison of scattered light intensities with OD_600_ values for three different *S. cerevisiae* strains with data collected from dilution series of stationary phase cells suspensions. **b** Comparison of scattered light intensities with OD_600_ values of dilution series data from stationary cells and sampled data from exponentially growing cells for *S. cerevisiae* CEN.PK2-1C. Scatterplots represent data measured in several parallel correlation experiments. *Line plots* represent the respective correlation function fit as described in the “Methods” section

Another interesting observation is the strain specific decrease in the scattered light intensity at the transition from sugar fermentation to respiratory ethanol consumption within the diauxic shift (Figs. 2, 3). This shift seems to cause morphological or cell size related changes that influence the scattering intensity to various degrees depending on the investigated strain (see also Fig. 5). While the growth curve of Ethanol Red exhibits a relatively fast and small signal decrease, scattered light intensities of BY4741 are decreasing much stronger and over a longer period of time. CEN.PK2-1C and W303-1A exhibit only very slight backscattering changes with minimal signal decreases over longer time periods (>6 h).

### Correlation of OD_600_ and scattering signal in shake flasks

In order to determine absolute biomass concentrations from scattering signals it is required to correlate offline biomass concentration measurements and the obtained scattering intensities. This correlation was investigated with different *S. cerevisiae* strains for exponentially growing cells as well as diluting stationary cell suspensions to prove the general applicability of CGQ’s scattering based biomass estimation in shake flasks and to identify its limitations.

Data collected via the dilution series approach (see “Correlation experiments” section) are shown in Fig. 5a for the three *S. cerevisiae* strains CEN.PK2-1C, W303-1A and Ethanol Red. The curves exhibit a strictly monotonic nonlinear correlation, which can be accurately described by the mixed exponential-sigmoid-linear model function (“Calculation basics—correlation backscatter vs. OD600”, “Calculation basics—growth experiments” sections). Even though all investigated strains belong to the same species, considerable strain-to-strain differences were observed for the correlation data. This might be attributed to differences in cell wall and cell interior composition as well as to cell size and morphology. Interestingly, W303-1A and Ethanol Red curves were highly similar up to an OD_600_ of 7 compared to the CEN.PK2-1C curve. W303-1A exhibited the lowest scattering. For W303, Kokina et al. [23] recently described changes in the correlation of cell count and OD_600_ due to increased cell swelling of aged cultures. As the cell from the presented correlation experiments originate from >72 h old cultures, the lower scattering of W303 cells could be explained by increased cell size and thus reduced backscattering [50].

Significant correlation differences were obtained between the dilution series approach and the growth approach (Fig. 5b). Within the range of OD_600_ = 1.5–3.5 the backscattered light intensity is much weaker for the exponentially growing cells than for the stationary cells. At an OD_600_ of about four the two correlation curves intersect. This behaviour can be explained by growth phase related changes of *S. cerevisiae’s* cell size. From Mie-theory of light scattering by homogeneous spheres it is known that backscattering intensities increase with decreasing particle volume, as shown by Latimer and Pyle [50] for various cell volumes and scattering angles. Hence it must be concluded from the correlation data sets that, within the range of OD_600_ = 1.5–3.5, the scattering particles from the growth approach are considerably larger than those from the dilution series approach, which is indeed caused by the mother-daughter-cell-aggregates from budding during the exponential growth, while the stationary phase as well as the slow growth on ethanol is dominated by single, non-aggregated and thus stronger backscattering cells, as clearly shown by the microscopic images in Fig. 2. Remaining differences at an OD_600_ > 4 could be due to fermentatively formed ethanol, which has been shown to induce significant changes to *S. cerevisiae’s* cell volume (size reductions >20 %) [51], as well as cell wall thickness, roughness and composition [52].

While morphologic, cell size, growth phase and even measurement technique related phenomena may introduce considerable variance into the direct correlation of backscattered light intensities and OD_600_ or other biomass estimates, these correlation data are still useful to calculate desired biomass values from backscatter readings as measured by the CGQ system. This is exemplarily shown in Fig. 2, where exponential growth phase (dark blue) and stationary growth phase (light blue) correlations were used to calculate OD_600_ from the CGQ’s backscatter readings and where offline data (yellow) were collected on a completely different UV–VIS-spectrometer. The good agreement between offline and online data could be reached by correlation data refinement within the CGQuant software. For this purpose, two additional offline OD_600_ (yellow squares with purple edges) values were entered into the software, the cell density after inoculation and after 31.5 h of fermentation.

### Resolution and reproducibility of light scattering based biomass estimation in shake flasks

**Fig. 6:**
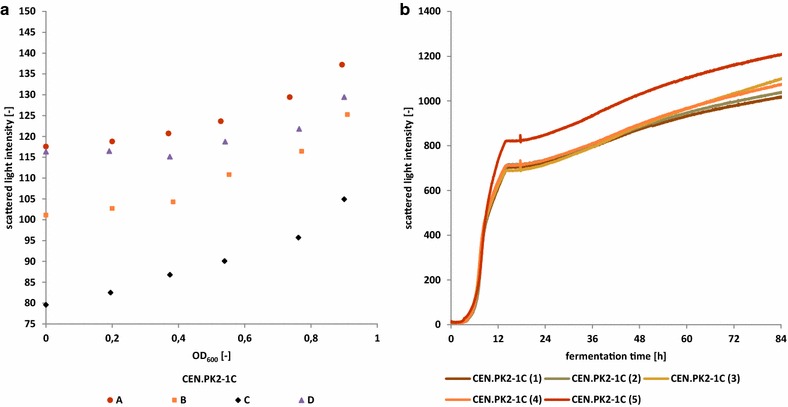
Reproducibility and resolution of scattered light measurements. **a** Comparison of four single correlation measurements for *S. cerevisiae* CEN.PK2-1C from dilution series of concentrated stationary phase cells. Chart **a** depicts a detailed view of the OD_600_ range 0–1. Chart **b** shows the comparison of five single growth experiments of *S. cerevisiae* CEN.PK2-1C on SCD2%. The final OD_600_ of all cultures was in a range of 5–6

Under optimal conditions, the CGQ’s backscatter readings provided sufficient resolution for biomass estimation even below an OD_600_ of 0.2, as exemplarily depicted in Fig. 6a. Positive correlations of scattering signal and OD_600_ could be observed for all other data sets at the latest from OD_600_ = 0.4 onwards. Throughout all measurements during this study, a high reproducibility (Fig. 6b) was observed for both, correlation and growth experiments. The baseline signal (see Fig. 4 BY4741, W303 1A) was highly stable with a background noise of 1 % and a signal drift smaller than 0.02 % per hour with respect to the average baseline signal.

The limits of resolution and technical reproducibility during backscatter monitoring are mainly introduced by the comparability of real shake flasks and their positioning in the spring clamps. Shake flask bottoms may exhibit different thicknesses, inhomogeneities, scratches and unevenness, all influencing the optical path. Spring clamps may show various degrees of stability and deformation, which can influence the positioning of the flask and thus the liquid distribution above the sensor. Many of these effectors are only recognisable at very low biomass concentrations, but under rarely observed worst-case conditions lens-like scaling effects can be observed as exemplarily shown in Fig. 6b for CEN.PK2-1C(5). However, algorithms have been implemented into the CGQuant software allowing for the elimination or reduction of such external factors.

## Conclusion and outlook

Besides widely used OD600 measurements, cell dry/wet weight or counting numbers of viable/dead cells of total cell amount per volume, scattered light intensity measurement is an additional tool for absolute biomass determination [53]. The latter technique has been implemented in the cell growth quantifier (CGQ) in an innovative dynamic approach, which, based on high speed data acquisition, exhibited high signal resolution and smoothness accompanied by low background noise and signal drift. Out of all currently existing solutions for non-invasive biomass monitoring in shake flasks, its comparably small size, high parallelisability and dynamic range, real-time data availability and its applicability under almost any cultivation condition in combination with an intuitive software made it the optimal choice for an efficiently parallelisable, triplicate based growth evaluation.

Online biomass data were collected for four *S. cerevisiae* strains grown on synthetic complete medium with various carbon sources. The CGQ derived biomass data were in good agreement with offline OD_600_ values with a nonlinear strain- and growth-phase-dependent correlation of backscatter readings and OD_600_. The growth curves allowed for the identification of typical bioprocess related phenomena, i.e. diauxic growth as described by the Crabtree-effect, pre-culture dependent lag-phase differences and oxygen limitation. A detailed investigation of those or comparable phenomena is hardly achievable with the currently most often used manual biomass measurement approach, thus making online biomass monitoring a valuable tool for bioprocess analysis in shake flasks.

The ability to detect even smallest differences in growth rates as well as the relation of cell size and morphology to the observed backscattered light intensity may be utilized in subsequent investigations regarding the derivation of morphologic data from the scattering behaviour of different eu- and prokaryotic species and strains. Future investigations may furthermore exploit the CGQ raw data to evaluate bioprocess related changes of fluid dynamic regimes or different viscosities, which are important in the fermentation of mycelia forming fungi or biopolymer-producing cells.

In conclusion, highly parallelisable online monitoring of shake flask cultures on the basis of light scattering is a promising technique and can be beneficial in a broad range of applications, from automated process monitoring and quality control to data and knowledge driven bioprocess development and optimisation. Especially the latter can strongly increase efficiency and liberate resources, as other process analysis and control tasks, i.e. HPLC, mass spectrometry, general protein analytics, expression induction and feeding, can be applied with high accuracy regarding cultivation time and progress under the guidance of online growth and growth rate curves.

## Methods

### Biomass monitoring principles

The CGQ performs biomass estimation on the basis of backscattered light measurements through the shake flask bottom (Fig. 1a, b). A LED with a central wavelength of 520 nm irradiates light into the fermentation broth. Depending on the present biomass concentration, a certain percentage of that light is scattered back by the cells and detected by a photodiode, which converts the photons into a weak electric current. This current is converted and amplified into a voltage and subsequently digitized, yielding a single backscatter reading. During one measurement cycle of about 1–2 s, millions of single backscatter readings are collected at a dynamic measurement frequency >500 kHz, which results in a raw data series (Fig. 1c). The periodic signal shape originates solely from the shaking induced liquid level changes above the sensor, as the cell density and thus the scattering particle concentration can be assumed to be constant over one measurement cycle. Based on the high raw data density and resolution within one measurement series, several nonlinear analysis approaches are applied to extract robust, reliable and smooth backscatter readings that can be used for biomass estimation in the range of OD_600_ < 0.2 to OD_600_ > 50 over a broad variety of different shaking and fermentation conditions.

The complete measuring electronics and firmware are bundled into a small CGQ sensor plate, which is clicked into the spring clamp prior to mounting the shake flask on the shaker (Fig. 1d). Each shake flask is covered by a darkening shield that prevents interference of environmental light with the sensitive measurement process. Up to 16 CGQ sensor plates are connected to one base station serving as a communication manager with the computer and as a power supplier to the CGQ sensor plates. Visualization, data analysis and comparison are carried out using the CGQuant software, which is also used for the collection of calibration/correlation data.

### Cultivation conditions

Cultivations were performed at defined synthetic complete media (SC) (similar to [27]), consisting of the following compounds: 0.17 % (w/v) Difco™ Yeast Nitrogen Base w/o aminoacids and ammonium sulfate, 0.5 % (w/v) ammonium sulfate, 11.2 mg/L adenine (0.083 mM), 38.4 mg/L^−1^l-arginine (0.221 mM), 19.2 mg/L l-histidine (0.124 mM), 57.6 mg/L l-isoleucine (0.439 mM), 57.6 mg/L l-leucine (0439 mM), 57.6 mg/L l-lysine monohydrate (0.394 mM), 38.4 mg/L l-methionine (0.258 mM), 48 mg/L l-phenylalanine (0.291 mM), 57.6 mg/L l-threonine (0.484 mM), 19.2 mg/L l-tryptophan (0.093 mM), 14.4 mg/L l-tyrosine (0.080 mM), 19.2 mg/L uracil (0.171 mM), 57.6 mg/L l-valine (0.492 mM). As sole carbon sources, 2 % (w/v) of d-glucose, ethanol, d-galactose and 1 % (w/v) of maltose, indicated as SCD2%, SCE2%, SCGal2% and SCM1%, respectively, were added to the media. The pH value was adjusted to 6.3 using potassium hydroxide. Pre-cultivations were performed at complete media (YEPD2%) [27] (In reference [27] mentioned as YEP glucose). As sole carbon source 2 % d-glucose was used.

### Yeast strains

The *S. cerevisiae* strains used throughout this work are listed in Table 1 together with relevant information regarding genotype, ploidity and source.

**Table 1:** *Saccharomyces cerevisiae* strains used in this work

Strains	Relevant genotype	Ploidy	Source
CEN.PK2-1C	MATa; his3∆1; leu2-3_112; ura3-52; trp1-289; MAL2-8c; SUC2	Haploid	EUROSCARF, Frankfurt a.M., Germany
W303-1A	MATa leu2-3112 trp1-1 can1-100 ura3-1 ade2-1 his3-11,15	Haploid	EUROSCARF, Frankfurt a.M., Germany
BY4741	MATa his3Δ1 leu2Δ0 LYS2 met15Δ0 ura3Δ0	Haploid	EUROSCARF, Frankfurt a.M., Germany
Ethanol Red	Industrial bioethanol production strain, *MATa/α*	Diploid	Fermentis, division of S. I. Lesaffre, Lille, France

### Growth experiments

Yeast strains were pre-grown on 100 mL YEPD2% media [27] in 500 mL flasks and harvested at OD_600_ of 1–2 through centrifugation (4500 rpm, 3 min). After an additional washing step with ddH_2_O, cultivations were grown on 50 mL defined synthetic complete media in 300 mL flasks (start OD_600_ of 0.2). Biomass was monitored online and non-invasively by cell growth quantifier (aquila biolabs GmbH). Cultivations were performed at 30 °C and 180 rpm. Measurements were stopped by non-detectable growth for 72 h or by reaching stationary phase.

#### Correlation experiments

Two different types of correlation experiments were conducted using the calibration module of the CGQuant software:

1. Dilution series approach
  Cells of different *S. cerevisiae* strains were grown on SCD2% medium and harvested at the early stationary phase. The cells were centrifuged and washed with fresh media, yielding defined stock solutions that were subsequently utilized for the standard CGQuant calibration procedure. This procedure started with filling the shake flask with pure medium followed by an iteratively repeated process of:
  measuring the backscatter intensity
  sampling 1 mL of liquid
  measuring the OD_600_ of that sample offline
  adding 1 mL of concentrated cell stock solution.
2. Growth approach
  CEN.PK2-1C cells were grown on SCD2% and at certain time points backscatter intensities were collected. Subsequent samples of 1 mL were taken out and the offline OD_600_ was determined. One millilitre of fresh medium was then added to the shake flask to ensure a constant filling volume.

For OD_600_ measurements samples were manually diluted in order to maintain linear correlation. OD_600_ values were determined in triplicates by Ultrospec 2100 pro from Amersham Biosciences. Correlation data were fitted to the bellow described sigmoid-linear correlation model using the calibration module of the CGQuant software.

For the growth assay shown in Fig. 2, offline-samples (1 mL) were collected from two identically inoculated flasks. First, 100 µL were used to determine offline OD_600_ values with a UV-3100PC (VWR) UV–VIS-spectrometer. Single droplets of same samples were used to collect microscopic images at a magnification of 800× using a Bresser Trino microscope. Metabolites of remaining cell free supernatant were analysed by HPLC. Concentrations of glucose and ethanol were determined by UHPLC UltiMate3000 using a HyperREZ™ XP Carbohydrate H^+^ column (both ThermoFisher Scientific). The column was eluted with 5 mM sulfuric acid as mobile phase and a flow rate of 0.6 mL min^−1^ at 65 °C. Refractive index values were detected by RI-101 (Shodex).

#### Calculation basics—correlation backscatter vs. OD_600_

The correlation of backscatter intensities and offline OD_600_ was evaluated using an empirical nonlinear model function, which was fitted onto the data sets within and as implemented by the CGQuant software (aquila biolabs GmbH). While an analytic derivation of this model function on the basis of scattering physics is out of the scope of this publication, some explanatory information on its applicability will be provided in the following.

Various functions have been applied to model the correlation of backscatter intensities and offline biomass measures (OD_600_ or cell dry weight) in continuously shaken bioreactor systems. The simplest one has been proposed by Kensy et al. [10], formulating a purely linear correlation, given by

$$I_{s} = m \cdot X + n$$

with *I*_*s*_ the scattered light intensity, *X* the biomass concentration, *m* the slope and *n* the y-axis intercept. The authors state a wide application range of 0–50 g/L cell dry weight. However, there are considerable systematic differences observable between measured and modelled data for both investigated organisms (*H. polymorpha* and *E. coli*). Especially in the lower and intermediate biomass concentration range, the assumption of a purely linear correlation appears to be too imprecise. Ude et al. [54] as well as Schmidt-Hager et al. [8] proposed several different nonlinear model functions for correlation of backscatter intensities and offline biomass measures, such as polynomial, exponential and Bleasdale-Nelder functions. However, each organism under investigation required a different model type for optimal correlation description. In conclusion, a linear correlation appears to be applicable at higher biomass concentrations, while nonlinear models may describe the lower concentration range better.

Based on that conclusion, the model function applied in the present publication was developed as a combination of both, a linear and a shapeable nonlinear component, and given by

$$I_{s} = \left( {m \cdot X + n} \right) \cdot \frac{{p_{1} }}{{\left( {1 + p_{2} \cdot e^{{ - p_{3} \cdot X^{{p_{4} - p_{5} }} }} } \right)^{{p_{6} }} }}$$

with *I*_*s*_ the scattered light intensity, *X* the biomass concentration, *m* the slope and *n* the y-axis intercept of the linear correlation at higher cell densities and *p*_1–6_ the parameters of the exponential sigmoid function describing the low and intermediate cell densities. This equation describes adequately the observed correlations for a broad range of shaken bioprocesses, as it empirically takes into account the dominating optical effects during a light scattering measurement. A simplified scattering experiment with non-absorbing medium and cells and without multiple scattering events could be described by the linear correlation alone. With increasing biomass concentrations, the observable correlation approaches that linear model, as the efficient backscattering volume and thus the probability of a photon to be absorbed by medium or cell components is reduced. At lower biomass concentrations the efficient backscattering volume is much larger (and may initially be even larger than the available liquid volume), so that absorption path lengths and thus absorption probabilities are higher. Volumetric and absorptive effects introduce significant deviations from the linear correlation, which can be described as an exponential relationship [54]. This exponential component is, along with the linear component, set into a sigmoid context and extended by two additional exponent-parameters (*p*_4_*, p*_6_), yielding the above formulated function with a tuneable transition range from exponential to linear correlation of backscatter intensity and biomass concentration.

#### Calculation basics—growth experiments

Growth rates are calculated in CGQuant as *µ* according to the differential equation of microbial growth

$$\frac{\partial X}{\partial t} = \mu \cdot X$$

with *X* the biomass concentration, *t* the time and *µ* the growth rate. Solving this differential equation with respect to time *t* yields the exponential formulation of microbial growth, given by

$$X_{t} = X_{0} \cdot e^{\mu \cdot t}$$

with *X*_*t*_ the biomass concentration at time *t* and *X*_0_ the initial biomass. During a typical batch fermentation, even the growth rate *µ* is a function of time *t*. In reaction of cells to their environment (e.g. substrate or oxygen limitations, metabolic shifts, toxins or toxic metabolites and products, pH, stress, etc.) the growth rate *µ* changes continuously throughout the bioprocess. The assumption of exponential growth according to above equations with an almost constant growth rate *µ* therefore holds true only for very small time spans, where the environmental conditions can be regarded as constant. Combining the exponential growth equations for two time points *t*_1_*, t*_2_ yields

$$\frac{{X_{{t_{1} }} }}{{e^{{\mu \cdot t_{1} }} }} = \frac{{X_{{t_{2} }} }}{{e^{{\mu \cdot t_{2} }} }}$$

which can be rewritten to

$$\mu = \frac{{\ln \frac{{X_{{t_{2} }} }}{{X_{{t_{1} }} }}}}{{(t_{2} - t_{1} )}}$$

with *µ* being the average exponential growth rate over the time interval from *t*_1_ to *t*_2_. Based on this equation, CGQuant executes a modified weighted moving average algorithm that shifts the time interval through all collected cell density data, yielding continuous growth rate curves. By increasing the time interval, the growth rate curves can be smoothed within the CGQuant software. Smoothing levels of 200 (equals a time interval of 70 min) were applied to all recorded growth curves.

As backscatter readings are, with some exceptions (e.g. strong morphologic changes that massively influence the cells scattering behaviour), strictly monotonic functions of the biomass concentration or its equivalent descriptors (OD_600_, cell dry weight, cell count), that kind of growth rate calculation can be also applied to the backscatter signal directly and without any additional calibration, yielding apparent growth rates. While apparent growth rates may significantly differ from those of calibrated or offline determined real growth rates, they still contain a lot of information. A peak in a real growth rate curve will remain a peak in an apparent growth rate curve, thus allowing for the identification and location with respect to time of metabolic changes or other fermentation events.

In order to compensate baseline differences caused by shake flask bottom inhomogeneities, all collected datasets were corrected using the auto-offset function in CGQuant. This function can be applied whenever the initial biomass concentration of curves to be compared is similar.
